# Co-occurrence of Fatigue and Depression in People With Multiple Sclerosis: A Mini-Review

**DOI:** 10.3389/fneur.2021.817256

**Published:** 2022-02-15

**Authors:** Joanna Tarasiuk, Katarzyna Kapica-Topczewska, Agata Czarnowska, Monika Chorąży, Jan Kochanowicz, Alina Kułakowska

**Affiliations:** Department of Neurology, Medical University of Bialystok, Białystok, Poland

**Keywords:** multiple sclerosis, fatigue, depression, anhedonia, fatigue scales

## Abstract

Fatigue and depression are common conditions diagnosed in people with multiple sclerosis (MS). Fatigue defined as subjective lack of physical and/or mental energy is present in 35–97% of people with MS, who classify it as one of the most serious symptoms interfering with daily activities and influencing the quality of life. Depression is diagnosed in about 50% of people with MS. Since fatigue and depression frequently coexists, it may be quite hard to differentiate them. Primary fatigue and primary depression in MS are caused by inflammatory, oxidative/nitrosative, and neurodegenerative processes leading to demyelination, axonal damage, and brain atrophy. In people with MS and comorbid fatigue and/or depression there is reported increased serum and cerebrospinal fluid concentration of inflammatory mediators such as tumor necrosis factor, interleukins (IL-1a, IL-1b, IL-6), interferon γ and neopterin. Moreover, the brain atrophy of prefrontal, frontal, parietotemporal regions, thalamus, and basal ganglia was observed in people with MS with fatigue and/or depression. The secondary fatigue and secondary depression in people with MS may be caused by emotional factors, sleep disorders, pain, the coexistence of other diseases, and the use of medications. In some studies, the use of disease-modifying therapies positively influenced fatigue, probably by reducing the inflammatory response, which proves that fatigue and depression are closely related to immunological factors. In this mini-review, the pathogenesis, methods of evaluation and differentiation, and possible therapies for fatigue and depression in MS are discussed.

## Introduction

Fatigue and depression are very common conditions diagnosed in people with multiple sclerosis (MS). Fatigue is present in 35–97% of people with MS (1, 2). It is classified as one of the most serious symptoms interfering with daily activities and influencing the quality of life (QoL) (1–3). Fatigue is defined as a subjective lack of physical and/or mental energy. MS-related fatigue is divided into physical and cognitive (4). Physical fatigue, defined as a decline in motor performance during sustained muscle activity, is caused by physical exhaustion and results from muscle weakness. Cognitive fatigue is defined as a decline in performance during cognitive activity, which results from difficulty with concentration, memory loss, and emotional instability (4–8). Cognitive fatigue starts independently from the physical disability in the early stages of MS and may be present already in the prediagnostic phase of the disease (9, 10). Cognitive fatigue is one of the key factors resulting in a decreased QoL in all people with MS (8, 11–13). Depression is diagnosed in about 50% of people with MS (14). Depression itself can manifest with fatigue and symptoms of depression may be mistaken for fatigue making these conditions difficult to differentiate. Recent studies have identified a strong correlation between fatigue and depression. These conditions jointly affect more than half of people with MS (15).

The leading and common symptom of cognitive fatigue and depression is anhedonia defined as decreased motivation, a lack of positive affect, and the reduced ability to experience pleasure (14, 16–18). Anhedonia is caused by deficiency of neurotransmitters such as dopamine and serotonin, which leads to impairment in the functioning of mesocorticolimbic pathways projecting from the midbrain to the basal ganglia, the limbic system, and the prefrontal cortex. It results in disrupting the brain's reward and valence system (16, 18). The structural and functional alterations of mesocorticolimbic pathways have been confirmed in neuroimaging studies in people with MS suffering from fatigue and/or depression (18).

The frequent coexistence of fatigue and depression in people with MS suggests a common etiology of both conditions (19–21). Primary fatigue and primary depression in MS are most probably caused by inflammatory, oxidative/nitrosative, and neurodegenerative processes leading to demyelination, axonal damage, and brain atrophy (1). In people with MS and comorbid fatigue and/or depression there is reported increased serum and cerebrospinal fluid (CSF) concentration of proinflammatory cytokines, interleukins, interferon γ (IFNγ), and neopterin (1). Many studies have been also reported the brain atrophy of the prefrontal, frontal, parietotemporal region, thalamus, and basal ganglia (16, 22). The secondary causes of fatigue and depression are emotional stress, sleep disorders, pain, the coexistence of other diseases, and the use of some disease-modifying therapies (DMTs), e.g., interferon-β (22). The treatment of MSrelated fatigue and depression is still challenging. In some studies, the use of natalizumab, fingolimod, and glatiramer acetate positively influenced fatigue, probably by reducing the inflammatory response, which proves that fatigue and depression are related to immunological factors (16).

In the present mini-review, we provide and discuss the latest information on the pathogenesis, methods of evaluation and differentiation, and possible therapies for fatigue and depression in MS.

## Etiopathogenesis of Fatigue and Depression in MS

**Figure 1:**
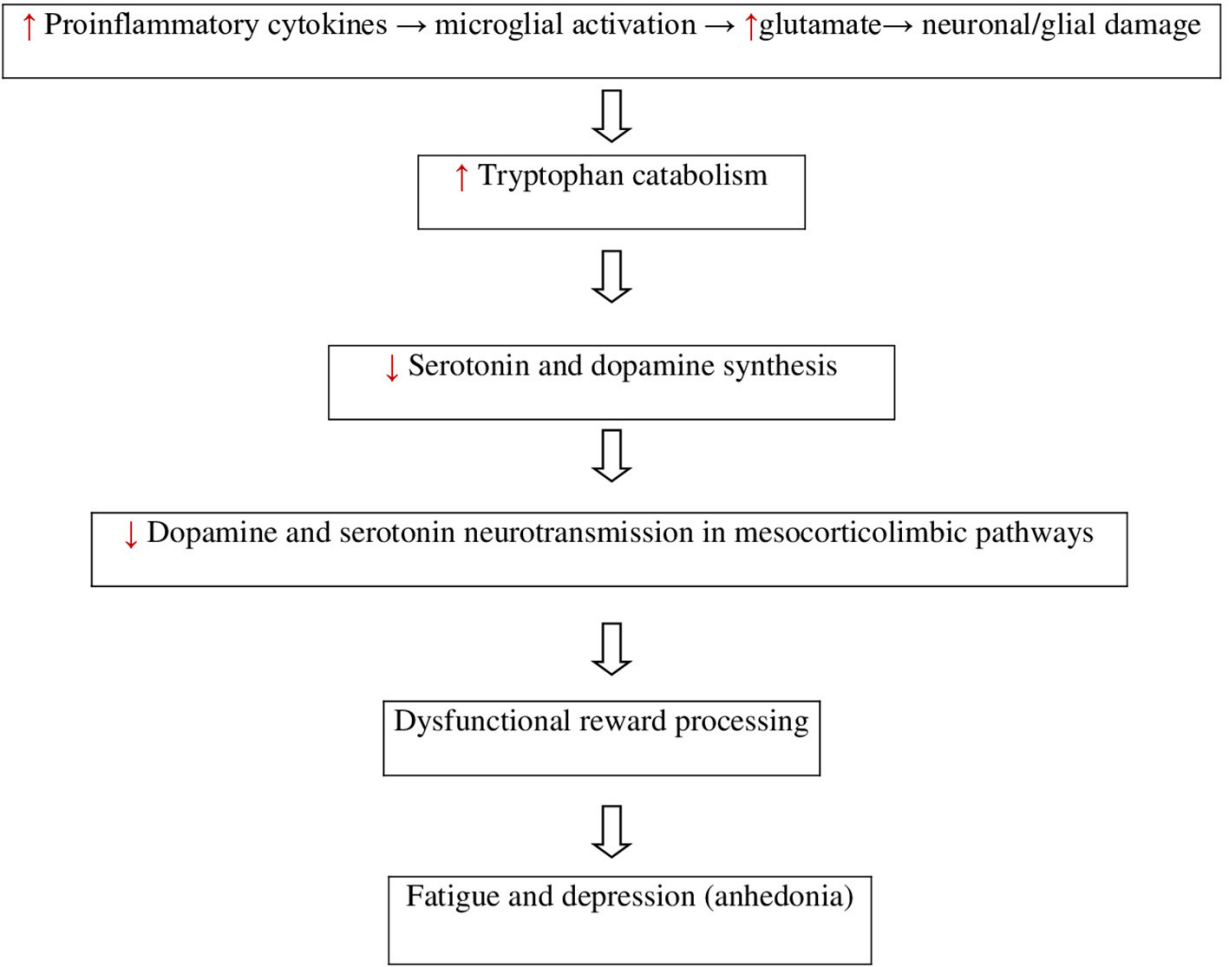
Neuroimmunological finding in MS-related fatigue and depression.

The neuroinflammatory process undergoing the pathogenesis of MS disturbs neural function and may result in fatigue and depression (Figure 1) (1–3, 15, 16). In the pathomechanisms of fatigue and depression in MS the crucial role play proinflammatory cytokines including tumor necrosis factor α (TNFα), interleukins (IL-1a, IL-1b, IL-2, IL-6), IFN-γ released by mitogen-stimulated peripheral blood lymphocytes, and neopterin produced by macrophages upon IFN-γ stimulation (23). The proinflammatory mediators lead to the induction of tryptophan catabolism. Tryptophan is a biochemical precursor for serotonin and kynurenine. The low level of these monoamines may lead to fatigue and depression (23). In people with MS with comorbid fatigue and/or depression, there are reported increased serum and CSF concentrations of the pro-inflammatory cytokines, such as interleukins (IL-1a, IL-1b, IL-2, IL-6), TNF-α and IFN-γ. The high concentrations of those pro-inflammatory mediators correlate directly with the level of fatigue and depression (1, 23–25). The proinflammatory cytokines in people with MS induce sickness behavior by disruption of dopamine and serotonin neurotransmission in mesocorticolimbic pathways connecting the midbrain with the basal ganglia, the limbic system, and the prefrontal cortex leading to dysfunctional reward processing and anhedonia (16, 18, 19, 26, 27). Pro-inflammatory cytokines disturb the synthesis of dopamine and serotonin by reducing the synaptic availability of amino acids precursor, disturbing their release, and increasing the reuptake of monoamines (27). The cytokines increase the metabolism of the serotonin precursor tryptophan *via* the alternative kynurenine pathway by inducing indoleamine 2,3 dioxygenase (IDO) (28). In addition, the cytokines decrease the availability of the co-factor tetrahydrobiopterin (BH4) limiting the turnover of the precursor phenylalanine and tyrosine and interfering with the formation of dopamine (29). The synaptic availability of serotonin and dopamine is reduced by decreased presynaptic release and increased activity of pro-inflammatory cytokines acting as reuptake transporters (30).

Recently, it has been shown that the microglia contribute to neurodegeneration by the production of neurotoxic metabolites such as quinolinic acid that maintains inflammation and neurodegeneration through excitotoxicity (16, 28). Increased glutamate levels in the CNS lead to overstimulation of glutamate receptors and neuronal and glial damage (31). Quinolinic acid stimulates release and inhibits the reuptake of glutamate from astrocytes as well as it is direct agonist binding to glutamate N-methyl-D aspartate (NMDA) receptors (32). Stimulation of extrasynaptic NMDA receptors by glutamate was reported to be associated with decreased expression of brain-derived neurotrophic factor (BDNF) and the induction of cell death. The pro-inflammatory cytokines contribute to excitotoxicity in the gray and white matter by hampering glutamate reuptake through astrocytes and oligodendrocytes (16, 31).

The neurodegeneration and decreased neurogenesis are also caused by oxidative and nitrosative stress (O&NS) ongoing in course of MS (1, 23, 33, 34). O&NS induces damage to membrane fatty acids and proteins, which results in the formation of anchorage neo-epitopes, exposed to an autoimmune response. The level of immunoglobulins M (IgM) against these epitopes (ex. palmitic, myristic, S-farnesyl-cysteine) was found to be increased in people with depression and fatigue. O&NS also leads to dysfunction of mitochondria, affects DNA expression, lowers antioxidant and omega-3 polyunsaturated fatty acid levels, and increases translocation of gram-negative bacteria. Evidence of the O&NS pathways shared by depression and fatigue may explain the common co-occurrence of these conditions in the course of MS (35).

In people with MS and comorbid fatigue and/or depression, there was also reported impairment of the hypothalamic-pituitary-adrenal (HPA) axis. The low cortisol and low dehydroepiandrosterone levels have been implicated in chronic fatigue and depression (22, 36, 37). It suggests a possible endocrine contribution to fatigue and depression. People with MS report increased energy after taking corticosteroids as treatment for MS relapse, which supports a possible positive hormonal influence of steroids on fatigue (22).

The etiopathogenesis of MS-related fatigue and depression is also involved serotonergic regulation *via* the brain serotonin transporters (SERT) (38). In people with MS the SERT regulation may be disturbed (38, 39). The SERT inhibitors, such as fluoxetine and sertraline have been reported to have neuroprotective effects in MS (38, 40). Hesse et al. have reported that serotonergic neurotransmission in people with MS is altered in limbic and paralimbic regions, the frontal cortex, which contributes to cognitive fatigue and depression in MS (38). People with MS and comorbid fatigue and/or depression have been reported to have low SERT availability in cortical and subcortical brain areas, limbic and paralimbic regions such as the cingulate cortex, hippocampal/parahippocampal, and insular (38).

## Anatomical Abnormalities in Fatigue and Depression in MS

Fatigue and depression in people with MS are associated with gray matter atrophy in the prefrontal cortex, the basal ganglia, the striatum, and the limbic system (16, 41–44). Many studies have shown decreased monoaminergic neurotransmission in frontostriatal and frontolimbic pathways in people with MS suffering from fatigue and depression (44–47). Roelcke et al. have shown in positron emission tomography study the decreased glucose metabolism in the basal ganglia and the prefrontal cortex. In turn, Finke et al. and Jaeger et al. have reported in the functional magnetic resonance imaging study the decreased functional connectivity between the ventral striatum, the amygdala, and the prefrontal cortex (48).

## Secondary Causes of Fatigue and/or Depression in MS

Fatigue and/or depression can be a psychological and emotional reaction to the lifestyle changes that occur when people are diagnosed with a chronic disease such as MS (49, 50). Fatigue and depression may be also caused by sleep disorders, pain, and the coexistence of other diseases. Sleep disorders are quite common in people with MS compared to the general population and may result from muscle spasticity and pain, emotional disturbances, nocturia, taking medications, and restless legs syndrome (RLS) (51). RLSis present in 30–50% of people with MS and deteriorates the sleep quality (52). The frequency of RLS increases with disability progression assessed by the Expanded Disability Status Score (EDSS) (52). In the course of MS dopaminergic diencephalospinal, and reticulospinal pathways projecting to the spinal cord may be damaged, which leads to RLS (53). People with MS presenting medullary lesions affecting respiratory centers may develop sleep breathing disorders such as central sleep apnea (54). The severity of disability assessed with EDSS also proportionally increases the risk of fatigue and depression (15, 55).

The use of some DMTs may also increase the risk of fatigue and depression (56). Fatigue and depression are reported more frequently in people treated with interferon-β, which causes side effects like flu-like symptoms resembling sickness behavior (57, 58). In some articles, depression is listed as a possible side effect of interferon-β (59, 60). On the contrary, there are several studies that have not found any relationship between DMT type and depression (61). There are still no conclusive data regarding DMTs influence on fatigue symptoms. Some publications raise the positive impact of natalizumab, fingolimod, and glatiramer acetate on fatigue and depression (60, 62–67). One of the theories is that antifatigue and antidepressive effectiveness of some DMTs may be related to the suppression of inflammatory pathways leading to depression (68, 69). However, a causal relationship between DMTs, especially T and B-cell depleting therapies, and the risk of depression remains to be shown. It is also important to consider the mode of administration of DMTs. Additional studies evaluating treatment satisfaction and quality of life of people with MS may shed light on the relation between treatment tolerability, mode of DMTs administration, and risk of fatigue and depression (70). There is a higher risk of DMTs discontinuation in people with MS and depression (60).

## Evaluation and Differential Diagnosis of Fatigue and Depression in People With MS

Fatigue and depression interfere with patients' daily activity and may lead to DMTs discontinuation. Therefore, there is a need for an early diagnosis and treatment. The assessment of fatigue is difficult and still challenging, as it requires objective measurement tools (5). Fatigue and depression may manifest with the same symptoms, like loss of motivation and anhedonia making these conditions difficult to differentiate. The fatigue in people with MS is classified on the basis of symptoms as physical, cognitive, and emotional. The fatigue symptoms are reduction in physical activity, problems with performing cognitive tasks, decreased concentration, memory disorders, executive difficulties, and a feeling of internal tension, anxiety, sadness (71).

The measurement of fatigue in the dimension of its perception is only subjective, while the objective measurement of fatigue may be assessed by analyzing the way cognitive and motor tasks are performed over time (27). In the subjective assessment of fatigue are used one- or multi-dimensional self-report scales in the form of questionnaires describing fatigue in terms of its occurrence (or not), severity, duration, and dimension (cognitive/physical). The one-dimensional tool is the Visual Analog Scale for Fatigue (VAS-F). The most commonly used multivariate scales are the short seven-point Fatigue Severity Scale (FSS) and the broader 21-point Modified Fatigue Impact Scale (MFIS). MFIS assesses the impact of fatigue on functioning in three dimensions: social, cognitive, and physical (72, 73). Another multidimensional self-report tool is the Fatigue Scale for Motor and Cognitive Function (FSMC), which assesses the occurrence and intensity of physical and cognitive fatigue on two 10-point scales (5). The objective measurement of fatigue in MS is based on quantitative and qualitative data obtained during the performance of the motor and cognitive tasks by patients. Physical fatigue is described in terms of a decrease in strength, energy, accuracy, or speed of performing the activity over time. In the case of cognitive tasks, the indicators of fatigue include the reduction of reaction time and accuracy during task performance (73).

The frequently comorbid depression in MS patients affects the occurrence of cognitive fatigue. The factor connecting both fatigue and depression is attention deficit (74, 75). Brenner and Piehl showed in their studies that depressive patients presented more severe symptoms of fatigue, which suggests that the onset of depression may be a predictor of fatigue and anxiety, and the onset of fatigue and anxiety may be a predictor of depression (76).

According to Penner et al. and Griffith and Zarrouf for depression is a typical depressed mood, hopelessness, loss of self-confidence and self-esteem, causeless self-reproaches or appropriate feelings of guilt, best functionality in the evening, patients usually attribute their illness to psychological factors (35), there is need for excessive sleep (hypersomnia) or early awakening (77). On the other hand for fatigue is typical hopeful and strong wish to recover, best functioning in the morning with a decrease during the day, patients take initiative while searching for treatment (35) and attribute reasons of fatigue for external factors, they may have difficulties to fall asleep and to maintain sleep resulting in decreased sleep quality (77).

In the differential diagnosis of fatigue and depression also should be performed laboratory testing for hematologic and metabolic conditions, such as thyroid studies, iron, 25- hydroxy vitamin D and vitamin B12 deficiency, ferritin, and folate levels (22).

## Therapeutic Approach to Fatigue and Depression in People With MS

Up to date, there is not enough evidence supporting the use of any medications for the treatment of MS-related fatigue (78). In clinical practice for fatigue treatment in people with MS are used amantadine, modafinil, and amphetamine-like stimulants (methylphenidate) (78). Amantadine is approved by the Food and Drug Administration (FDA) for treatment of influenza and Parkinson's disease and causes an increase in cholinergic and dopaminergic transmission. Modafinil is approved by the FDA for narcolepsy, shift-work sleep disorder, and obstructive sleep apnea with residual excessive sleepiness. Amantadine and modafinil have been tested in clinical trials for fatigue in people with MS, but their results have been conflicting (79, 80). Recently, Nourbakhsh et al. in a randomized, double-blind trial compared the efficacy, safety, and tolerability of amantadine, modafinil, methylphenidate, and placebo in people with MS-related fatigue. The results of its study have shown no the superiority of these drugs according to placebo in improving MS-related fatigue, which might have been influenced by comorbid depression and other diseases, MS subtype, the severity of the physical disability, or use of DMTs. However, in *post-hoc* analysis modafinil and methylphenidate might have a marginal, but clinically significant effect on fatigue in patients with excessive daytime sleepiness, which suggests that excessive daytime sleepiness may lead to fatigue in some people with MS (78).

In the treatment of fatigue and depression in people with MS are also used drugs enhancing monoamine neurotransmissions, such as selective serotonin and noradrenaline reuptake inhibitors and psychostimulants with dopaminergic effects (2, 19). Tricyclic antidepressants are effective in reducing clinical depression and improving sleep patterns and are reported beneficial for patients with chronic fatigue.

Recent studies show that non-pharmacological interventions, such as physical exercises and psychological therapy may reduce MS-related fatigue or depression more effectively than pharmacological medications (81, 82). Non-pharmacologic treatment of fatigue or depression includes cognitive-behavioral therapy (CBT), relaxation therapy, physical exercises and rehabilitation, resistance training, mindfulness, yoga, and tai chi, optimal diet, and appropriate sleep hygiene (81, 83, 84). CBT changes the dysfunctional and emphasizes more realistic cognitions, behaviors, and emotions that are responsible for fatigue or depression (82). Recent reports show that CBT has a positive effect on MS-related fatigue, however, this effect decreases with cessation of treatment (85).

The very important strategy in MS-related fatigue and/or depression management is self-management education (SME) (8, 86). This is a complex intervention combining the provision of information and behavior change techniques, to influence the way patients experience the disease (8). SME teaches patients how to cope with a disease‘s symptoms and enables helpful behaviors, habits, and routines. SME in people with MS reduces fatigue and improves QoL (8). According to Lorig and Holman, SME solves medical, emotional, and role management problems, helps make decisions, and taking action, resources utilization, forms a patient/health care provider partnership (86). The medical management of fatigue is based on symptoms reduction and treatment. Emotional management influences thoughts, beliefs, and behaviors related to cognitive fatigue and is approached by CBT and relaxation exercises. The coping with daily tasks and duties is provided by occupational therapy (OT), which teaches conservation and management strategies, e.g., daily activity schedules, occupational balance or workload, and environment adaptation (8).

## Conclusions

The prevalence of fatigue and depression in people with MS is very high. Fatigue and depression in people with MS have multifactorial etiology, such as inflammatory and neurodegenerative processes, oxidative/nitrosative stress, leading to axonal damage, and demyelination, as well as brain atrophy of prefrontal, frontal, parietotemporal regions, thalamus, and basal ganglia. The inflammatory etiology of fatigue and depression in MS was supported by evidence of increased serum and CSF concentration of inflammatory mediators such as TNF, interleukins (IL-1a, IL-1b, IL-6), IFNγ, and neopterin. The secondary fatigue and secondary depression in people with MS may be caused by emotional factors, sleep disorders, pain, the coexistence of other diseases and, the use of some medication. There is not enough evidence supporting the use of any medications for the treatment of MS-related fatigue. In the treatment of depression and fatigue in people with MS are frequently used drugs enhancing monoamine neurotransmission and non-pharmacologic methods, such as CBT, relaxation therapy, OT, and physical rehabilitation. Recently, progress has been made in evaluating CBT or OT, but evaluation of the patient‘s education, which teaches self-management skills, helps to cope with disease-related fatigue, and leads to improvement of QoL, is lacking. The interventions, such as self-management education are difficult to evaluate, because of many possible outcome dimensions, instruments, and measurement time-points.

Therefore, there is needed for further researches on neuroimmune interactions, inflammatory biomarkers, the HPA-axis, and neurotransmitters in the pathogenesis of fatigue and depression in people with MS. There is also a high need for the development of new assessment tools for fatigue diagnostics and its differentiation with depression, the assessment of pharmacological and non-pharmacological treatment effectiveness, and the influence of DMTs on the development and course of MS-related fatigue and depression.

## Author Contributions

JT and AK contributed to conception and design of the manuscript and wrote the first draft of the manuscript. JT, AK, KK-T, AC, MC, and JK wrote sections of the manuscript. All authors contributed to manuscript revision, read, and approved the submitted version.

## Conflict of Interest

The authors declare that the research was conducted in the absence of any commercial or financial relationships that could be construed as a potential conflict of interest.

## Publisher's Note

All claims expressed in this article are solely those of the authors and do not necessarily represent those of their affiliated organizations, or those of the publisher, the editors and the reviewers. Any product that may be evaluated in this article, or claim that may be made by its manufacturer, is not guaranteed or endorsed by the publisher.

## References

[1] Pokryszko-Dragan A Frydecka I Kosmaczewska A Ciszak L Bilińska M Gruszka E. Stimulated peripheral production of interferon-gamma is related to fatigue and depression in multiple sclerosis. Clin Neurol Neurosurg. (2012) 114:1153–8. 10.1016/j.clineuro.2012.02.048 22425464

[2] Penner IK Paul F. Fatigue as a symptom or comorbidity of neurological diseases. Nat Rev Neurol. (2017) 13:662–75. 10.1038/nrneurol.2017.117 29027539

[3] Bakshi R. Fatigue associated with multiple sclerosis: diagnosis, impact and management. Mult Scler. (2003) 9:219–27. 10.1191/1352458503ms904oa 12814166

[4] David Ruban S Christina Hilt C Petersen T. Quality of life in multiple sclerosis: The differential impact of motor and cognitive fatigue. Mult Scler J Exp Transl Clin. (2021) 7:2055217321996040. 10.1177/2055217321996040 33708414 PMC7907948

[5] Penner IK Raselli C Stöcklin M Opwis K Kappos L Calabrese P. The fatigue scale for motor and cognitive functions (FSMC): validation of a new instrument to assess multiple sclerosis-related fatigue. Mult Scler. (2009) 15:1509–17. 10.1177/1352458509348519 19995840

[6] Linnhoff S Fiene M Heinze HJ Zaehle T. Cognitive fatigue in multiple sclerosis: an objective approach to diagnosis and treatment by transcranial electrical stimulation. Brain Sci. (2019) 9:100. 10.3390/brainsci9050100 31052593 PMC6562441

[7] Rottoli M La Gioia S Frigeni B Barcella V. Pathophysiology, assessment and management of multiple sclerosis fatigue: an update. Expert Rev Neurother. (2017) 17:373–9. 10.1080/14737175.2017.1247695 27728987

[8] Hersche R Roser K Weise A Michel G Barbero M. Fatigue self-management education in persons with disease-related fatigue: a comprehensive review of the effectiveness on fatigue and quality of life. Patient Educ Couns. (2021). 10.1016/j.pec.2021.09.016. [Epub ahead of print]. 34561143

[9] Minden SL Frankel D Hadden L Perloffp J Srinath KP Hoaglin DC. The Sonya Slifka longitudinal multiple sclerosis study: methods and sample characteristics. Mult Scler. (2006) 12:24–38. 10.1191/135248506ms1262oa 16459717

[10] Disanto G Zecca C MacLachlan S Sacco R Handunnetthi L Meier UC. Prodromal symptoms of multiple sclerosis in primary care. Ann Neurol. (2018) 83:1162–73. 10.1002/ana.25247 29740872

[11] Kobelt G Eriksson J Phillips G Berg J. The burden of multiple sclerosis 2015: methods of data collection, assessment and analysis of costs, quality of life and symptoms. Mult Scler. (2017) 23(Suppl. 2):4–16. 10.1177/1352458517708097 28643592

[12] Olsson T Achiron A Alfredsson L Berger T Brassat D Chan A. Anti-JC virus antibody prevalence in a multinational multiple sclerosis cohort. Mult Scler. (2013) 19:1533–8. 10.1177/1352458513477925 23459571

[13] Berger T Kobelt G Berg J Capsa D Gannedahl M Platform EMS. New insights into the burden and costs of multiple sclerosis in Europe: results for Austria. Mult Scler. (2017) 23(Suppl. 2):17–28. 10.1177/1352458517708099 28643599

[14] Feinstein A Magalhaes S Richard JF Audet B Moore C. The link between multiple sclerosis and depression. Nat Rev Neurol. (2014) 10:507–17. 10.1038/nrneurol.2014.139 25112509

[15] Patrick E Christodoulou C Krupp LB Consortium NYSM. Longitudinal correlates of fatigue in multiple sclerosis. Mult Scler. (2009) 15:258–61. 10.1177/1352458508097466 19181775

[16] Heitmann H Andlauer TFM Korn T Mühlau M Henningsen P Hemmer B. Fatigue, depression, and pain in multiple sclerosis: how neuroinflammation translates into dysfunctional reward processing and anhedonic symptoms. Mult Scler. (2020) 1352458520972279. 10.1177/1352458520972279. [Epub ahead of print]. 33179588 PMC9131410

[17] Husain M Roiser JP. Neuroscience of apathy and anhedonia: a transdiagnostic approach. Nat Rev Neurosci. (2018) 19:470–84. 10.1038/s41583-018-0029-9 29946157

[18] Swardfager W Rosenblat JD Benlamri M McIntyre RS. Mapping inflammation onto mood: inflammatory mediators of anhedonia. Neurosci Biobehav Rev. (2016) 64:148–66. 10.1016/j.neubiorev.2016.02.017 26915929

[19] Solaro C Gamberini G Masuccio FG. Depression in multiple sclerosis: epidemiology, aetiology, diagnosis and treatment. CNS Drugs. (2018) 32:117–33. 10.1007/s40263-018-0489-5 29417493

[20] Heitmann H Haller B Tiemann L Mühlau M Berthele A Tölle TR. Longitudinal prevalence and determinants of pain in multiple sclerosis: results from the German National Multiple Sclerosis Cohort study. Pain. (2020) 161:787–96. 10.1097/j.pain.0000000000001767 32197038

[21] Ayache SS Chalah MA. Fatigue and affective manifestations in multiple sclerosis-A cluster approach. Brain Sci. (2019) 10:10. 10.3390/brainsci10010010 31877878 PMC7017318

[22] Braley TJ Chervin RD. Fatigue in multiple sclerosis: mechanisms, evaluation, and treatment. Sleep. (2010) 33:1061–7. 10.1093/sleep/33.8.1061 20815187 PMC2910465

[23] Maes M Twisk FN Kubera M Ringel K. Evidence for inflammation and activation of cell- mediated immunity in Myalgic Encephalomyelitis/Chronic Fatigue Syndrome (ME/CFS): increased interleukin-1, tumor necrosis factor-α, PMN-elastase, lysozyme and neopterin. J Affect Disord. (2012) 136:933–9. 10.1016/j.jad.2011.09.004 21975140

[24] Brenner P Granqvist M Königsson J Al Nimer F Piehl F Jokinen J. Depression and fatigue in multiple sclerosis: relation to exposure to violence and cerebrospinal fluid immunomarkers. Psychoneuroendocrinology. (2018) 89:53–8. 10.1016/j.psyneuen.2018.01.002 29324301

[25] Malekzadeh A Van de Geer-Peeters W De Groot V Teunissen CE Beckerman H Group T-AS. Fatigue in patients with multiple sclerosis: is it related to pro- and anti-inflammatory cytokines? Dis Markers. (2015) 2015:758314. 10.1155/2015/758314 25722532 PMC4313513

[26] Pardini M Capello E Krueger F Mancardi G Uccelli A. Reward responsiveness and fatigue in multiple sclerosis. Mult Scler. (2013) 19:233–40. 10.1177/1352458512451509 22723570

[27] Manjaly ZM Harrison NA Critchley HD Do CT Stefanics G Wenderoth N. Pathophysiological and cognitive mechanisms of fatigue in multiple sclerosis. J Neurol Neurosurg Psychiatry. (2019) 90:642–51. 10.1136/jnnp-2018-320050 30683707 PMC6581095

[28] Raison CL Dantzer R Kelley KW Lawson MA Woolwine BJ Vogt G. CSF concentrations of brain tryptophan and kynurenines during immune stimulation with IFN-alpha: relationship to CNS immune responses and depression. Mol Psychiatry. (2010) 15:393–403. 10.1038/mp.2009.116 19918244 PMC2844942

[29] Felger JC Li L Marvar PJ Woolwine BJ Harrison DG Raison CL. Tyrosine metabolism during interferon-alpha administration: association with fatigue and CSF dopamine concentrations. Brain Behav Immun. (2013) 31:153–60. 10.1016/j.bbi.2012.10.010 23072726 PMC3578984

[30] Capuron L Pagnoni G Drake DF Woolwine BJ Spivey JR Crowe RJ. Dopaminergic mechanisms of reduced basal ganglia responses to hedonic reward during interferon alfa administration. Arch Gen Psychiatry. (2012) 69:1044–53. 10.1001/archgenpsychiatry.2011.2094 23026954 PMC3640298

[31] Korn T Magnus T Jung S. Autoantigen specific T cells inhibit glutamate uptake in astrocytes by decreasing expression of astrocytic glutamate transporter GLAST: a mechanism mediated by tumor necrosis factor-alpha. FASEB J. (2005) 19:1878–80. 10.1096/fj.05-3748fje 16123171

[32] Tavares RG Tasca CI Santos CE Alves LB Porciúncula LO Emanuelli T. Quinolinic acid stimulates synaptosomal glutamate release and inhibits glutamate uptake into astrocytes. Neurochem Int. (2002) 40:621–7. 10.1016/S0197-0186(01)00133-4 11900857

[33] Maes M. An intriguing and hitherto unexplained co-occurrence: depression and chronic fatigue syndrome are manifestations of shared inflammatory, oxidative and nitrosative (IO&NS) pathways. Prog Neuropsychopharmacol Biol Psychiatry. (2011) 35:784–94. 10.1016/j.pnpbp.2010.06.023 20609377

[34] Maes M Mihaylova I Kubera M Leunis JC Geffard M. IgM-mediated autoimmune responses directed against multiple neoepitopes in depression: new pathways that underpin the inflammatory and neuroprogressive pathophysiology. J Affect Disord. (2011) 135:414–8. 10.1016/j.jad.2011.08.023 21930301

[35] Penner IK Bechtel N Raselli C Stöcklin M Opwis K Kappos L. Fatigue in multiple sclerosis: relation to depression, physical impairment, personality and action control. Mult Scler. (2007) 13:1161–7. 10.1177/1352458507079267 17967844

[36] Cleare AJ. The neuroendocrinology of chronic fatigue syndrome. Endocr Rev. (2003) 24:236–52. 10.1210/er.2002-0014 12700181

[37] Gottschalk M Kümpfel T Flachenecker P Uhr M Trenkwalder C Holsboer F. Fatigue and regulation of the hypothalamo-pituitary-adrenal axis in multiple sclerosis. Arch Neurol. (2005) 62:277–80. 10.1001/archneur.62.2.277 15710856

[38] Hesse S Moeller F Petroff D Lobsien D Luthardt J Regenthal R. Altered serotonin transporter availability in patients with multiple sclerosis. Eur J Nucl Med Mol Imaging. (2014) 41:827–35. 10.1007/s00259-013-2636-z 24562640

[39] Taler M Gil-Ad I Korob I Weizman A. The immunomodulatory effect of the antidepressant sertraline in an experimental autoimmune encephalomyelitis mouse model of multiple sclerosis. Neuroimmunomodulation. (2011) 18:117–22. 10.1159/000321634 21088435

[40] Solaro C Bergamaschi R Rezzani C Mueller M Trabucco E Bargiggia V. Duloxetine is effective in treating depression in multiple sclerosis patients: an open-label multicenter study. Clin Neuropharmacol. (2013) 36:114–6. 10.1097/WNF.0b013e3182996400 23783007

[41] Palotai M Guttmann CR. Brain anatomical correlates of fatigue in multiple sclerosis. Mult Scler. (2020) 26:751–64. 10.1177/1352458519876032 31536461

[42] Schmaal L Veltman DJ van Erp TG Sämann PG Frodl T Jahanshad N. Subcortical brain alterations in major depressive disorder: findings from the ENIGMA major depressive disorder working group. Mol Psychiatry. (2016) 21:806–12. 10.1038/mp.2015.69 26122586 PMC4879183

[43] Calabrese M Rinaldi F Grossi P Mattisi I Bernardi V Favaretto A. Basal ganglia and frontal/parietal cortical atrophy is associated with fatigue in relapsing-remitting multiple sclerosis. Mult Scler. (2010) 16:1220–8. 10.1177/1352458510376405 20670981

[44] Nigro S Passamonti L Riccelli R Toschi N Rocca F Valentino P. Structural 'connectomic' alterations in the limbic system of multiple sclerosis patients with major depression. Mult Scler. (2015) 21:1003–12. 10.1177/1352458514558474 25533294

[45] Pardini M Bonzano L Mancardi GL Roccatagliata L. Frontal networks play a role in fatigue perception in multiple sclerosis. Behav Neurosci. (2010) 124:329–36. 10.1037/a0019585 20528076

[46] Palotai M Cavallari M Koubiyr I Morales Pinzon A Nazeri A Healy BC. Microstructural fronto-striatal and temporo-insular alterations are associated with fatigue in patients with multiple sclerosis independent of white matter lesion load and depression. Mult Scler. (2020) 26:1708–18. 10.1177/1352458519869185 31418637

[47] Feinstein A O'Connor P Akbar N Moradzadeh L Scott CJ Lobaugh NJ. Diffusion tensor imaging abnormalities in depressed multiple sclerosis patients. Mult Scler. (2010) 16:189–96. 10.1177/1352458509355461 20007425

[48] Finke C Schlichting J Papazoglou S Scheel M Freing A Soemmer C. Altered basal ganglia functional connectivity in multiple sclerosis patients with fatigue. Mult Scler. (2015) 21:925–34. 10.1177/1352458514555784 25392321

[49] Wallin MT Wilken JA Turner AP Williams RM Kane R. Depression and multiple sclerosis: review of a lethal combination. J Rehabil Res Dev. (2006) 43:45–62. 10.1682/JRRD.2004.09.0117 16847771

[50] Kirzinger SS Jones J Siegwald A Crush AB. Relationship between disease-modifying therapy and depression in multiple sclerosis. Int J MS Care. (2013) 15:107–12. 10.7224/1537-2073.2012-036 24453772 PMC3883027

[51] Chesson A Hartse K Anderson WM Davila D Johnson S Littner M. Practice parameters for the evaluation of chronic insomnia. An American academy of sleep medicine report standards of practice Committee of the American Academy of Sleep Medicine. Sleep. (2000) 23:237–41. 10.1093/sleep/23.2.1k 10737341

[52] Manconi M Ferini-Strambi L Filippi M Bonanni E Iudice A Murri L. Multicenter case- control study on restless legs syndrome in multiple sclerosis: the REMS study. Sleep. (2008) 31:944–52. 10.5665/sleep/31.7.944 18655317 PMC2491510

[53] Clemens S Rye D Hochman S. Restless legs syndrome: revisiting the dopamine hypothesis from the spinal cord perspective. Neurology. (2006) 67:125–30. 10.1212/01.wnl.0000223316.53428.c9 16832090

[54] Auer RN Rowlands CG Perry SF Remmers JE. Multiple sclerosis with medullary plaques and fatal sleep apnea (Ondine's curse). Clin Neuropathol. (1996) 15:101–5. 8925593

[55] Téllez N Río J Tintoré M Nos C Galán I Montalban X. Does the Modified Fatigue Impact Scale offer a more comprehensive assessment of fatigue in MS? Mult Scler. (2005) 11:198–202. 10.1191/1352458505ms1148oa 15794395

[56] Hadjimichael O Vollmer T Oleen-Burkey M. Sclerosis NARCoM. Fatigue characteristics in multiple sclerosis: the North American Research Committee on Multiple Sclerosis (NARCOMS) survey. Health Qual Life Outcomes. (2008) 6:100. 10.1186/1477-7525-6-100 19014588 PMC2596785

[57] Giovannoni G Southam E Waubant E. Systematic review of disease-modifying therapies to assess unmet needs in multiple sclerosis: tolerability and adherence. Mult Scler. (2012) 18:932–46. 10.1177/1352458511433302 22249762

[58] Alba Palé L León Caballero J Samsó Buxareu B Salgado Serrano P Pérez Solà V. Systematic review of depression in patients with multiple sclerosis and its relationship to interferonβ treatment. Mult Scler Relat Disord. (2017) 17:138–43. 10.1016/j.msard.2017.07.008 29055445

[59] Janssens AC van Doorn PA de Boer JB Kalkers NF van der Meche FG Passchier J. Anxiety and depression influence the relation between disability status and quality of life in multiple sclerosis. Mult Scler. (2003) 9:397–403. 10.1191/1352458503ms930oa 12926846

[60] Longinetti E Frisell T Englund S Reutfors J Fang F Piehl F. Risk of depression in multiple sclerosis across disease-modifying therapies. Mult Scler. (2021) 13524585211031128. 10.1177/13524585211031128. [Epub ahead of print]. 34264143 PMC8961249

[61] Feinstein A. Multiple sclerosis, disease modifying treatments and depression: a critical methodological review. Mult Scler. (2000) 6:343–8. 10.1177/135245850000600509 11064445

[62] Svenningsson A Falk E Celius EG Fuchs S Schreiber K Berkö S. Natalizumab treatment reduces fatigue in multiple sclerosis. Results from the TYNERGY trial; a study in the real life setting. PLoS ONE. (2013) 8:e58643. 10.1371/journal.pone.0058643 23555589 PMC3605436

[63] Penner IK Sivertsdotter EC Celius EG Fuchs S Schreiber K Berkö S. Improvement in fatigue during natalizumab treatment is linked to improvement in depression and day-time sleepiness. Front Neurol. (2015) 6:18. 10.3389/fneur.2015.00018 25755648 PMC4337367

[64] Hunter SF Agius M Miller DM Cutter G Barbato L McCague K. Impact of a switch to fingolimod on depressive symptoms in patients with relapsing multiple sclerosis: an analysis from the EPOC (Evaluate Patient OutComes) trial. J Neurol Sci. (2016) 365:190–8. 10.1016/j.jns.2016.03.024 27206905

[65] Kunkel A Fischer M Faiss J Dähne D Köhler W Faiss JH. Impact of natalizumab treatment on fatigue, mood, and aspects of cognition in relapsing-remitting multiple sclerosis. Front Neurol. (2015) 6:97. 10.3389/fneur.2015.00097 26029156 PMC4426783

[66] Lang C Reiss C Mäurer M. Natalizumab may improve cognition and mood in multiple sclerosis. Eur Neurol. (2012) 67:162–6. 10.1159/000334722 22269396

[67] Metz LM Patten SB Archibald CJ Bakker JI Harris CJ Patry DG. The effect of immunomodulatory treatment on multiple sclerosis fatigue. J Neurol Neurosurg Psychiatry. (2004) 75:1045–7. 10.1136/jnnp.2002.007724 15201369 PMC1739126

[68] Miller AH Raison CL. The role of inflammation in depression: from evolutionary imperative to modern treatment target. Nat Rev Immunol. (2016) 16:22–34. 10.1038/nri.2015.5 26711676 PMC5542678

[69] Robertson MJ Schacterle RS Mackin GA Wilson SN Bloomingdale KL Ritz J. Lymphocyte subset differences in patients with chronic fatigue syndrome, multiple sclerosis and major depression. Clin Exp Immunol. (2005) 141:326–32. 10.1111/j.1365-2249.2005.02833.x 15996197 PMC1809442

[70] Tarrants M Oleen-Burkey M Castelli-Haley J Lage MJ. The impact of comorbid depression on adherence to therapy for multiple sclerosis. Mult Scler Int. (2011) 2011:271321. 10.1155/2011/271321 22096632 PMC3196992

[71] Berard JA Bowman M Atkins HL Freedman MS Walker LA. Cognitive fatigue in individuals with multiple sclerosis undergoing immunoablative therapy and hematopoietic stem cell transplantation. J Neurol Sci. (2014) 336:132–7. 10.1016/j.jns.2013.10.023 24189209

[72] Mills RJ Young CA Pallant JF Tennant A. Rasch analysis of the modified fatigue impact scale (MFIS) in multiple sclerosis. J Neurol Neurosurg Psychiatry. (2010) 81:1049–51. 10.1136/jnnp.2008.151340 20547635

[73] Rudroff T Kindred JH Ketelhut NB. Fatigue in multiple sclerosis: misconceptions and future research directions. Front Neurol. (2016) 7:122. 10.3389/fneur.2016.00122 27531990 PMC4969300

[74] Niino M Mifune N Kohriyama T Mori M Ohashi T Kawachi I. Apathy/depression, but not subjective fatigue, is related with cognitive dysfunction in patients with multiple sclerosis. BMC Neurol. (2014) 14:3. 10.1186/1471-2377-14-3 24393373 PMC3884018

[75] Sundgren M Maurex L Wahlin Å Piehl F Brismar T. Cognitive impairment has a strong relation to nonsomatic symptoms of depression in relapsing-remitting multiple sclerosis. Arch Clin Neuropsychol. (2013) 28:144–55. 10.1093/arclin/acs113 23291310

[76] Brenner P Piehl F. Fatigue and depression in multiple sclerosis: pharmacological and non- pharmacological interventions. Acta Neurol Scand. (2016) 134(Suppl. 200):47–54. 10.1111/ane.12648 27580906

[77] Griffith JP Zarrouf FA. A systematic review of chronic fatigue syndrome: don't assume it's depression. Prim Care Companion J Clin Psychiatry. (2008) 10:120–8. 10.4088/PCC.v10n0206 18458765 PMC2292451

[78] Nourbakhsh B Revirajan N Morris B Cordano C Creasman J Manguinao M. Safety and efficacy of amantadine, modafinil, and methylphenidate for fatigue in multiple sclerosis: a randomised, placebo-controlled, crossover, double-blind trial. Lancet Neurol. (2021) 20:38–48. 10.1016/S1474-4422(20)30354-9 33242419 PMC7772747

[79] Ledinek AH Sajko MC Rot U. Evaluating the effects of amantadin, modafinil and acetyl-L- carnitine on fatigue in multiple sclerosis–result of a pilot randomized, blind study. Clin Neurol Neurosurg. (2013) 115(Suppl. 1):S86–9. 10.1016/j.clineuro.2013.09.029 24321164

[80] Sheng P Hou L Wang X Huang C Yu M Han X. Efficacy of modafinil on fatigue and excessive daytime sleepiness associated with neurological disorders: a systematic review and meta- analysis. PLoS ONE. (2013) 8:e81802. 10.1371/journal.pone.0081802 24312590 PMC3849275

[81] Khan F Amatya B Galea M. Management of fatigue in persons with multiple sclerosis. Front Neurol. (2014) 5:177. 10.3389/fneur.2014.00177 25309504 PMC4163985

[82] van den Akker LE Beckerman H Collette EH Twisk JW Bleijenberg G Dekker J. Cognitive behavioral therapy positively affects fatigue in patients with multiple sclerosis: results of a randomized controlled trial. Mult Scler. (2017) 23:1542–53. 10.1177/1352458517709361 28528567

[83] van Kessel K Moss-Morris R Willoughby E Chalder T Johnson MH Robinson E. randomized controlled trial of cognitive behavior therapy for multiple sclerosis fatigue. Psychosom Med. (2008) 70:205–13. 10.1097/PSY.0b013e3181643065 18256342

[84] Smith C Hale L Olson K Schneiders AG. How does exercise influence fatigue in people with multiple sclerosis? Disabil Rehabil. (2009) 31:685–92. 10.1080/09638280802273473 18841515

[85] van den Akker LE Beckerman H Collette EH Eijssen IC Dekker J de Groot V. Effectiveness of cognitive behavioral therapy for the treatment of fatigue in patients with multiple sclerosis: a systematic review and meta-analysis. J Psychosom Res. (2016) 90:33–42. 10.1016/j.jpsychores.2016.09.002 27772557

[86] Lorig KR Holman H. Self-management education: history, definition, outcomes, and mechanisms. Ann Behav Med. (2003) 26:1–7. 10.1207/S15324796ABM2601_01 12867348

